# Comparison, Analysis, and Molecular Dynamics Simulations of Structures of a Viral Protein Modeled Using Various Computational Tools

**DOI:** 10.3390/bioengineering10091004

**Published:** 2023-08-24

**Authors:** Hemalatha Mani, Chun-Chun Chang, Hao-Jen Hsu, Chin-Hao Yang, Jui-Hung Yen, Je-Wen Liou

**Affiliations:** 1 Institute of Medical Sciences, Tzu Chi University, Hualien 97004, Taiwan; 2 Department of Laboratory Medicine, Hualien Tzu Chi Hospital, Buddhist Tzu Chi Medical Foundation, Hualien 97004, Taiwan; 3 Department of Laboratory Medicine and Biotechnology, Tzu Chi University, Hualien 97004, Taiwan; 4 Department of Biomedical Sciences and Engineering, Tzu Chi University, Hualien 97004, Taiwan; 5 Department of Biochemistry, School of Medicine, Tzu Chi University, Hualien 97004, Taiwan; 6 Department of Molecular Biology and Human Genetics, Tzu Chi University, Hualien 97004, Taiwan

**Keywords:** homology modeling, AF2, Robetta-RoseTTAFold, trRosetta, MOE, I-TASSER, MD simulation, HCV core protein

## Abstract

The structural analysis of proteins is a major domain of biomedical research. Such analysis requires resolved three-dimensional structures of proteins. Advancements in computer technology have led to progress in biomedical research. In silico prediction and modeling approaches have facilitated the construction of protein structures, with or without structural templates. In this study, we used three neural network-based de novo modeling approaches—AlphaFold2 (AF2), Robetta-RoseTTAFold (Robetta), and transform-restrained Rosetta (trRosetta)—and two template-based tools—the Molecular Operating Environment (MOE) and iterative threading assembly refinement (I-TASSER)—to construct the structure of a viral capsid protein, hepatitis C virus core protein (HCVcp), whose structure have not been fully resolved by laboratory techniques. Templates with sufficient sequence identity for the homology modeling of complete HCVcp are currently unavailable. Therefore, we performed domain-based homology modeling for MOE simulations. The templates for each domain were obtained through sequence-based searches on NCBI and the Protein Data Bank. Then, the modeled domains were assembled to construct the complete structure of HCVcp. The full-length structure and two truncated forms modeled using various computational tools were compared. Molecular dynamics (MD) simulations were performed to refine the structures. The root mean square deviation of backbone atoms, root mean square fluctuation of Cα atoms, and radius of gyration were calculated to monitor structural changes and convergence in the simulations. The model quality was evaluated through ERRAT and phi–psi plot analysis. In terms of the initial prediction for protein modeling, Robetta and trRosetta outperformed AF2. Regarding template-based tools, MOE outperformed I-TASSER. MD simulations resulted in compactly folded protein structures, which were of good quality and theoretically accurate. Thus, the predicted structures of certain proteins must be refined to obtain reliable structural models. MD simulation is a promising tool for this purpose.

## 1. Introduction

Deep neural network-based approaches have facilitated studies on in silico protein structure prediction and protein–protein interactions. These approaches can help to predict the structures of a wide array of proteins and have been suggested in many cases with an accuracy similar to that of X-ray crystallography, nuclear magnetic resonance (NMR) spectroscopy, and cryogenic electron microscopy [1]. Sequence-based prediction of the three-dimensional (3D) structures of proteins has been a long-standing challenge. Several deep learning-based structure prediction tools have recently been demonstrated to outperform the traditional approaches for protein structure prediction [2,3,4]. AlphaFold (DeepMind) [5,6] is a powerful artificial intelligence tool that predicts the structures of proteins on the basis of their amino acid (AA) sequences. According to the Critical Assessment of Structural Prediction (CASP), the aforementioned tool exhibits a high accuracy [7,8,9]. Neural network-based algorithms are typically trained using supervised data obtained from the Protein Data Bank (PDB) [6,10,11]. Robetta [12] is a three-track deep neural network-based prediction interface with an accuracy that is close to that of AlphaFold2 (AF2) according to the results of CASP. The network considers the patterns of AA sequences and the interactions between AA residues to predict the 3D structures of proteins [12,13]. Transform-restrained Rosetta (trRosetta) [14] is a residual convolutional server that adopts a deep neural network-based approach to predict inter-residue geometries; the predicted geometries are transformed as restraints to guide structure prediction through direct energy minimization. In contrast to other approaches, trRosetta can predict geometries such as inter-residue distances and orientations [14].

A traditional method for in silico structural modeling is template-based homology modeling, in which suitable laboratory-derived resolved protein structures are used as templates for structure construction. The guidelines for generating credible models have been described previously [15]. Structural templates with >40% sequence identity to (and coverage of) target proteins are required for reliable modeling [16]. Normally, multisequence alignments are performed using sequence similarity search tools, such as the PDB basic local alignment search tool (BLAST), to identify tertiary structural templates. Unlike traditional methods used for identifying templates for homology modeling, iterative threading assembly refinement (I-TASSER) [17,18], an automatic template-based platform, automatically identifies suitable structural templates from the PDB by adopting a multiple threading approach. I-TASSER predicts protein structures based on a sequence-to-structure-to-function-based paradigm, and the models are assembled through iterative fragment simulations [17,19]. In CASP experiments, this tool has exhibited an excellent performance in predicting protein structures [18,20,21].

The hepatitis C virus (HCV) core protein (HCVcp) is a protein that assembles into the HCV capsid, which encloses and protects the viral genome [22,23,24,25]. In addition to its principal role, HCVcp alters host inflammatory responses to facilitate viral evasion from the host immune response and the establishment of persistent liver infection [26]. HCVcp exhibits both proinflammatory and anti-inflammatory activities [26]. Through its anti-inflammatory and proinflammatory activities, HCVcp facilitates viral survival and disease (hepatitis) development, respectively. HCVcp targets various compartments of HCV host cells [26,27,28]. Evidence suggests that HCVcp directly interacts with Toll-like receptors on host cells [29], which play key roles in mediating host inflammatory responses [30]. Prolonged inflammation leads to chronic hepatitis, liver cirrhosis, hepatocellular carcinoma, and extrahepatic HCV-related diseases [26]. The full-length sequence of HCVcp comprises 191 AAs and can be divided into three domains. Domain 1 (N-terminal AA 1 to AA 116 [31]) is the most hydrophilic HCVcp domain involved in viral genomic RNA binding [22], and this domain is responsible for viral capsid formation [32]. This domain also interacts with host cellular proteins [33,34,35]. Domain 2 (AA 117 to AA 177 [31]) is highly hydrophobic and possibly mediates the interactions of HCVcp with host lipids, lipid droplets, and membranes [22]. Domain 3 (AA 178 to AA 191 [31]) is extremely hydrophobic, enhances intracellular lipid accumulation, and is associated with hepatic steatosis [36,37]. The 3D structure of this viral protein is required for the comprehensive analyses of the interactions between HCVcp and host proteins/receptors. Such analyses facilitate the development of effective strategies for the prevention and treatment of HCV-related disorders. However, to the best of our knowledge, resolved tertiary structures of HCVcp are still unavailable, rendering it difficult to perform structure-based investigations of the interactions between HCVcp and host proteins. In this study, we constructed 3D structures of HCVcp by using deep neural network-based de novo structure prediction methods—AF2, Robetta, and trRosetta—and the automated template-based tool I-TASSER. In addition, suitable templates were identified through BLAST-based searches. Homology modeling of the HCVcp structure was performed using the Molecular Operating Environment (MOE). The structures were modeled using different computational tools and were then compared and analyzed. Molecular dynamics (MD) simulations of the modeled structures were also performed for structure refinement. Our findings provide key insights into the applications and efficiencies of in silico structural modeling methods for constructing protein structures, which are useful for structure-based biomedical research.

## 2. Materials and Methods

### 2.1. Amino Acid Sequence of HCVcp

The AA sequence of HCVcp used in this study was from the HCV genotype 1a isolate H77 [38] (NCBI reference sequence: YP_009709861; https://www.ncbi.nlm.nih.gov/protein/YP_009709861, access date: 18 June 2023). The sequence is identical to that with the UniProt IDs: A0A077D151_9HEPC and B3TKY8_9HEPC.

### 2.2. Prediction of the Secondary Structures of HCVcp

The secondary structures of HCVcp were predicted using PSIPRED (http://bioinf.cs.ucl.ac.uk/psipred/, access date: 11 April 2023) [39,40].

### 2.3. Structural Modeling of HCVcp

The structures of HCVcp Domain 1 (HCVcp 116) and Domains 1 and 2 (HCVcp 177), as well as that of the full-length protein (HCVcp 191), were modeled using online platforms or built-in software. The following tools were used for this purpose:

For the AF2-based prediction of the HCVcp structure, we used AlphaFold colab notebook (version 2.1.0; GitHub; https://colab.research.google.com/github/deepmind/alphafold/blob/main/notebooks/AlphaFold.ipynb#scrollTo=XUo6foMQxwS2, access date: 30 September 2022). This colab notebook does not require templates for structural modeling; the prediction is based solely on true de novo structure prediction [6].

For the Robetta-based prediction of the protein structure, the sequences were submitted to the Robetta-RoseTTAFold web server (https://Robetta-RoseTTAFold.bakerlab.org/, access date: 1 October 2022). The RoseTTAFold algorithm was used for generating the structural model. The accuracy and reliability of the predictions were continually evaluated (Continuous Automated Model Evaluation web server) [12].

For trRosetta-based prediction of the protein structure, we used the relevant web platform (https://yanglab.nankai.edu.cn/trRosetta/, access date: 18 October 2022). AA sequences were submitted to the platform. A deep residual neural network-based algorithm was used to predict inter-residue distances and orientations. The resulting 3D models were finalized through direct energy minimization performed using smooth restraints [14,41,42].

For MOE simulations, the BLAST (National Center for Biotechnology Information; https://blast.ncbi.nlm.nih.gov/Blast.cgi, access date: 27 October 2022) [43] was used to search and identify templates with sufficient sequence identity/similarity to the target protein. Then, the identified templates were aligned to the target protein sequences. Homology modeling was performed using MOE (Chemical Computing Group, Montreal, Quebec, Canada) to construct protein structures from the AA sequences.

The HCVcp structure was also modeled by I-TASSER (https://zhanggroup.org/I-TASSER/, access date: 21 September 2022), which is a hierarchical tool for predicting protein structures. This tool automatically identifies templates from the PDB through a multiple threading approach; full-length atomic models are constructed through iterative template-based fragment assembly simulations.

### 2.4. Visualization and Analysis of the Predicted Models

MOE version 2020.09 (license manager version 11.17.1) was used for the visualization, representation, and analysis of the predicted protein structures. In addition, this tool was used for generating the phi–psi plots, which were used to analyze the geometry of the predicted models and examine them for unusual or unreasonable features.

ERRAT analysis [44] was performed to analyze structural models; for this, the SAVES server (version 6.0; https://saves.mbi.ucla.edu/, access date: 3 January 2023) was used. We also used ProSA-web (https://prosa.services.came.sbg.ac.at/prosa.php, access date: 3 January 2023) [45] for structural evaluations.

### 2.5. MD Simulations

After structure preparations using MOE, MD simulations were performed using GROMACS (version 5.1.4) with a GROMOS 54A7 force field [46] for topology generation. The MD simulation protocols used in this study followed those indicated in our previous research [47,48]. The GROMOS force field is used in MD simulations for biomolecular systems such as peptides and proteins, nucleic acids, carbohydrates, and lipids at ambient temperatures and pressures of liquids, crystals, and solutions. The GROMOS 54A7 force field is a modified force field in which the stability of proteins has been tested and found to be in excellent agreement with a range of primary experimental data [49]. In this study, the three-point single point charge (SPC) water [50,51,52] was applied as the solvent, with coordinates from a pre-equilibrated box spc216.gro [51]. These coordinates can also be applied for other three-point water models, since the small differences between the models will be eliminated with a short equilibration [53]. The proteins were placed in the defined solvated water box. The periodic boundary conditions were set by commanding the image distance cutoff. During the simulation, Berendsen temperature and pressure coupling were applied. For temperature coupling, a Berendsen thermostat was used to maintain the system at a reference temperature of 310 K. The temperature coupling time constant was set to 0.2 ps. For pressure coupling, Berendsen exponential relaxation pressure coupling was enabled with a time constant of 1.5 ps. The integration time step (dt) was set at 0.002 ps for each run.

Energy minimization in GROMACS was performed using steepest descent and conjugate gradients, as suggested per protocol [54]. After energy minimization, position restrains were applied to achieve thermal equilibrium in order to ensure unrestricted simulation and avoid uneven solvent molecule distribution. The energy minimization was performed using step-by-step position restrains for temperature, pressure, and velocity normalization. This is to avoid drastic rearrangements of critical parts of the protein during equilibration. Followed by addition of solvent molecules, Na and Cl ions (concentration of 0.15 M) were added to the box. Each simulation was run for 200 ns. Details for all simulations in this study are listed in Supplementary Table S2. Solvated water boxes containing initial HCVcp model structures for MD simulations are illustrated in Supplementary Figures S2–S6. All MD simulation trajectory files were analyzed using the GROMACS built-in tools.

The RMSD of backbone atoms was calculated through the least-square fitting of the query structure to the reference structure; this was performed using the gmx rms command (*t*_2_ = 0) [55].

(1) $$RMSD\left(t_{1},t_{2}\right)={\left[\frac{1}{M}\overset{N}{\underset{i=1}{\sum }}m_{i}\|r_{i}\left(t_{1}\right)-r_{i}\left(t_{2}\right){\|}^{2}\;\right]}^{\frac{1}{2}}$$

*M* = $\sum_{i=1}^{N}m_{i}$ and $r_{i}\left(t\right)$ is the position of atom *ⅰ* at time *t* after the least-square fitting of the query structure to the reference structure. The fitting was performed with respect to the backbones of the query and reference structures.

The Rg value was calculated using the gmx gyrate command to measure the rough compactness of the structure.

(2) $$R_{g}={\left(\frac{\sum_{i}^{0}\|r_{i}{\|}^{2}\;m_{i}{}^{o}}{\sum_{i}^{0}m_{i}}\right)}^{\frac{1}{2}}$$

where $m_{i}$ is the mass of atom *ⅰ* and $r_{i}$ is the position of atom *ⅰ* with respect to the center of mass of the molecule. The fitting was performed with respect to the structure of the main chain.

To calculate atomic fluctuations, the gmx RMSF command was used, which computes the standard deviations of atomic positions in the trajectory after fitting them to a reference frame.

(3) $${\mathrm{RMSF}}_{i}={\left[\frac{1}{T}\overset{T}{\underset{t_{j}=1}{\sum }}\|r_{i}\left(t_{1}\right)-r_{i}\left(t_{2}\right){\|}^{2}\;\right]}^{\frac{1}{2}}$$

where *T* is the time over which the displacement of particle *i* was averaged and $r_{i}\left(t_{2}\right)$ is the reference position of particle *i*. In this study, the RMSF values of Cα atoms were calculated, and the values were plotted versus residue number *i*. The RMSF of an atom *i* was computed as the time average, and no mass weighting was performed [56,57].

## 3. Results

### 3.1. HCVcp Models

**Figure 1:**
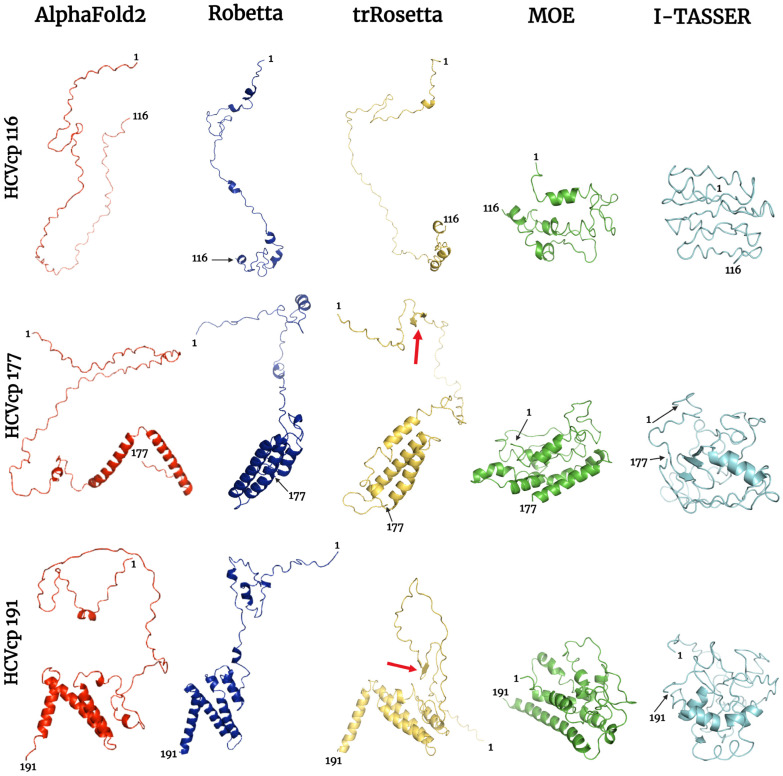
Tertiary structures of varying lengths of HCVcp (HCVcp 116, HCVcp 177, and HCVcp 191) predicted using AF2 (red), Robetta (blue), trRosetta (yellow), MOE (green), and I-TASSER (cyan). The positions of the first and last amino acids from the N-terminal of the protein are indicated. The red arrows indicate β-strands, which are also observed in the predicted secondary structures.

**Figure 2:**
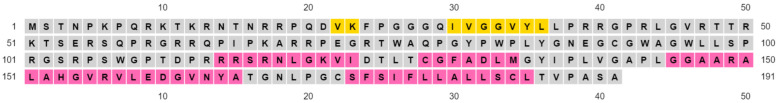
The AA sequence and secondary structure prediction of HCVcp 191 obtained by using PSIPRED 4.0. Numbers in the figure indicate the positions of amino acids from the N-terminal of the protein. Strands, helices, and coils are indicated with yellow, pink, and grey, respectively.

We first constructed HCVcp structures with varying lengths by using the deep neural network-based prediction tools AF2, Robetta, and trRosetta and the template-based modeling tools MOE and I-TASSER. The modeled structures were then compared and analyzed. To generate reliable results, structural templates with >40% sequence identity to the target protein are required for MOE homology modeling [58,59,60]. Unfortunately, for HCVcp, MOE could not obtain a template with sufficient sequence identity for structural modeling. Therefore, we performed homology modeling for each HCVcp domain and assembled the modeled domains to construct the complete structure of HCVcp. The PDB codes of the templates used in MOE simulations are presented in Supplementary Figure S1. I-TASSER automatically selects suitable templates for modeling. The templates selected by I-TASSER are listed in Supplementary Table S1. HCVcp structures of three lengths were modeled: HCVcp 116 (sequence: AA 1 to AA 116; comprising Domain 1 only), HCVcp 177 (sequence: AA 1 to AA 177; comprising Domains 1 and 2), and HCVcp 191 (the full-length HCVcp). The modeled structures are depicted in Figure 1. The structures modeled using neural network-based tools (AF2, Robetta, and trRosetta) were more extended, whereas those modeled using the template-based tools (MOE and I-TASSER) were more compact. We further predicted the secondary structures of HCVcp by using PSIPRED (http://bioinf.cs.ucl.ac.uk/psipred/, access date: 11 April 2023) [61]. The secondary structure prediction results are presented in Figure 2. The secondary structure predictions are considered highly accurate [62,63], and the prediction results in this study were compared with the modeled 3D structures. According to the secondary structure prediction results, HCVcp Domain 1 was largely unstructured and contained only two short β-strand structures; several clear α-helical structures were noted between AA 113 and the C-terminal of the protein (Domains 2 and 3). These prediction results agreed with the implications from an experimental study on HCVcp secondary structures using circular dichroism spectroscopy [64], in which Domain 1 was calculated to be largely unstructured, while the HCVcp with Domains 1 and 2 contained a large proportion of α-helical structures. As shown in Figure 1, the models of HCVcp 116 were mostly unstructured. However, helical structures were observed in the models constructed using Robetta, trRosetta, and MOE. None of the models contained the β-strand structures noted in secondary structure prediction. In all models of HCVcp 177, helical structures were observed near the C-terminals, which, to some extent, was consistent with the results of secondary structure prediction. However, β-strand structures were noted near the C-terminal of the I-TASSER-predicted model; this prediction varied from those of models predicted using the other tools and the results of secondary structure prediction. Among the modeling tools used, trRosetta generated a model with two short β-strand structures in the Domain 1 region; this model exhibited the highest level of agreement with the results of secondary structure prediction. The HCVcp 191 models predicted using the neural network-based tools were similar, with proximal C-terminal domains. The extent and orientations of α-helices present in these models were similar and highly consistent with the results of secondary structure prediction. By contrast, the HCVcp 191 models predicted using template-based tools varied considerably in terms of folding patterns. Among all predicted models of HCVcp 191, the model predicted using trRosetta exhibited the highest level of agreement with the results of secondary structure prediction.

### 3.2. Model Ranks and Structure Interpretations

**Figure 3:**
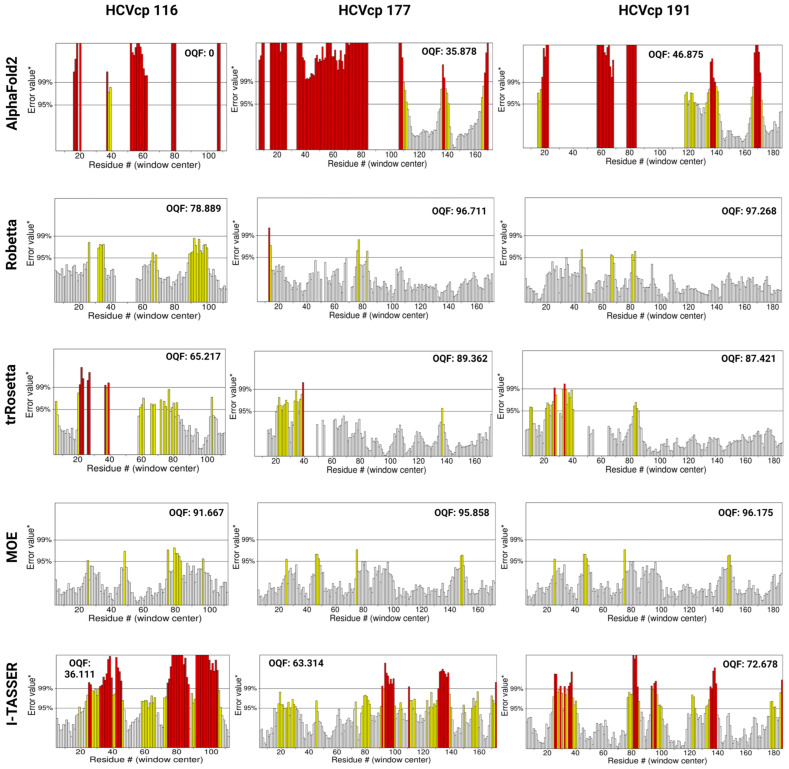
ERRAT plots for HCVcp structures with varying lengths (HCVcp 116, HCVcp 177, and HCVcp 191). The models were predicted using AF2, Robetta, trRosetta, MOE, and I-TASSER. The plots show two lines (on the error axis), which indicate the confidence level at which regions exceeding the error value can be rejected. The regions that can be rejected at the 95% confidence level are in yellow and those that can be rejected at the 99% confidence level (99% confidence in being erroneous) are in red. OQF, overall quality factor.

To evaluate the quality of the modeled structures, we analyzed parameters that are crucial for the quality control of tertiary structures. The ERRAT server (https://www.doe-mbi.ucla.edu/errat/, access date: 3 January 2023) validates protein models, including those obtained through X-ray crystallography, by using a quadratic error function to characterize noncovalent interactions [44]. The statistics of nonbonded interactions between various atoms are evaluated using the error function, which is calculated by comparing the aforementioned statistics with those of highly refined structures. This method highlights errors due to the random distributions of atoms, thus facilitating differentiation between the incorrect and correct regions of a predicted structure on the basis of atomic interactions. The ERRAT plots for the structural models constructed using different prediction tools are presented in Figure 3. According to the ERRAT plots, Robetta, trRosetta, and MOE generated structural models with high quality, with little to no erroneous regions in the structures. However, the models predicted using AF2 and I-TASSER had many erroneous regions with error values exceeding the 99% confidence intervals (marked as red bars in Figure 3), which indicated the poor quality of the models. The ERRAT server also provides an overall quality factor (OQF), which is a global score for identifying nonbonded atomic regions [44]. A higher ERRAT score indicates the better quality of the structure and thus the better reliability of the model. The OQFs for the modeled structures are also presented in Figure 3. For HCVcp 116, the model constructed using MOE had the best score (OQF: 91.667). For HCVcp 177, the models constructed using Robetta and MOE had the best scores (OQF: 96.711 and 95.858, respectively). Furthermore, for HCVcp 191, the models constructed using Robetta and MOE had the best scores (OQF: 97.268 and 96.175, respectively). On the basis of OQFs, all models constructed using AF2 and I-TASSER contained errors (i.e., low OQFs). Therefore, these poor-quality models required structural refinement.

### 3.3. Results of Stereochemical Analysis

**Figure 4:**
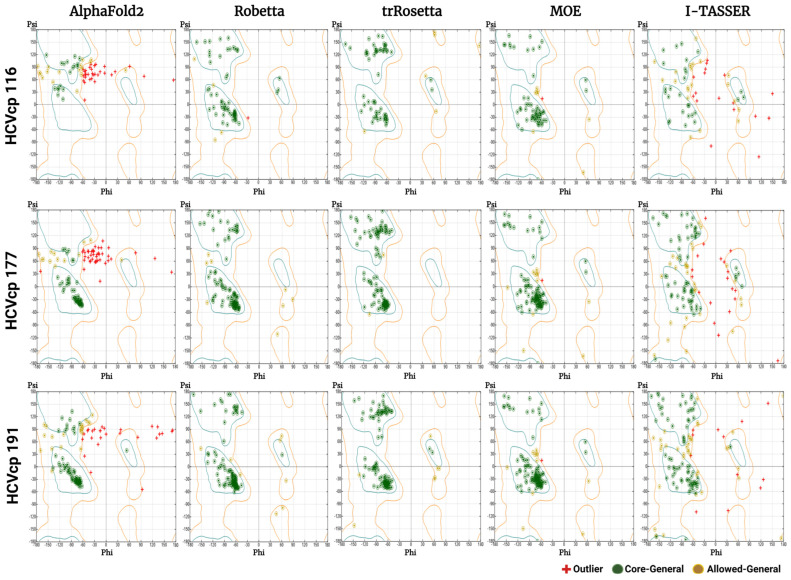
Phi–psi plots for the HCVcp 116, HCVcp 177, and HCVcp 191 models constructed using AF2, Robetta, trRosetta, MOE, and I-TASSER. The cyan outline corresponds to most favored regions (conformations with no steric hindrance). The orange outline corresponds to allowed regions. The regions outside the orange outline are disallowed regions (sterically forbidden regions).

Phi–psi plots (Ramachandran plots) can be used for the evaluation of predicted protein structures [65,66,67]. These plots serve as sensitive tools for assessing the torsion angles of protein backbones, thereby facilitating the unbiased validation of predicted protein structures. The plots show the phi and psi torsion angles for each residue in the main chain. In addition, the plots show favored regions corresponding to the conformations with dihedral angles which are typically observed in α-helices and β-sheets. In protein models, AA residues are distributed in the most favored regions (core regions), allowed regions, and disallowed regions. In an ideal protein structure, no residue should be present in disallowed (or outlier) regions. A higher distribution of residues in disallowed regions indicates a poorer quality of the protein model. Figure 4 shows the phi–psi plots of all models constructed in this study. The models constructed using AF2 and I-TASSER had clusters of residues in disallowed regions, suggesting the poor quality of the models. Models with poor quality should be remodeled or subjected to energy minimization to generate structural models with improved stereochemical properties. Notably, Robetta, trRosetta, and MOE generated models with a satisfactory quality according to the phi–psi plots. For structural refinement and energy minimization, we further performed MD simulations of all models constructed in this study.

### 3.4. Results of MD Simulations

**Figure 5:**
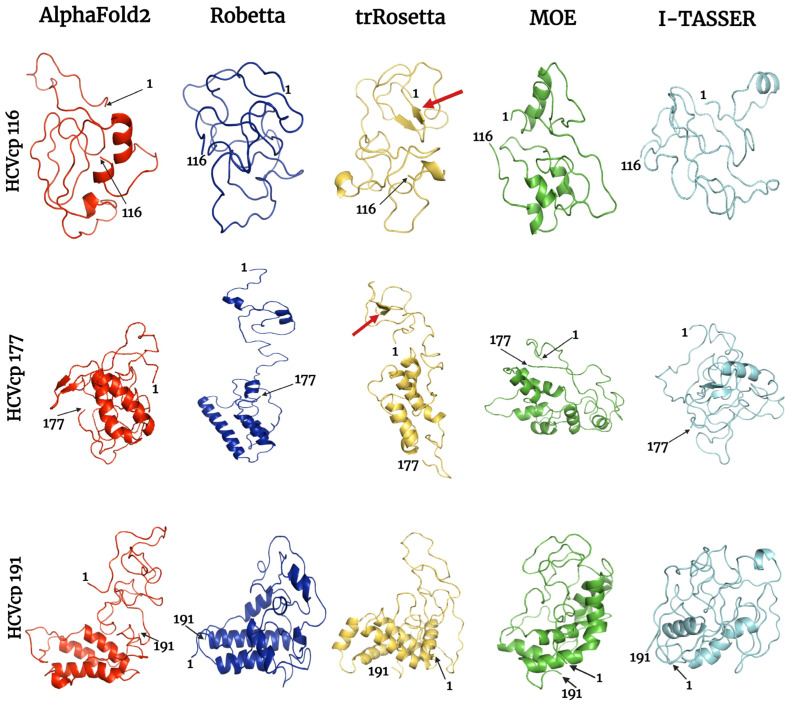
Final frames of the refined HCVcp 116, HCVcp 177, and HCVcp 191 models. The models were constructed using AF2 (red), Robetta (blue), trRosetta (yellow), MOE (green), and I-TASSER (cyan) and subsequently subjected to MD simulations. The positions of the first and last amino acids from the N-terminal of the protein are indicated. The red arrows indicate β-strands, which were also observed in the predicted secondary structures.

MD simulations can provide information on protein kinetics and thermodynamics [60] and can refine protein models [68]. In this study, we performed MD simulations to track conformational changes and protein folding until the constructed protein models reached a state with the lowest internal free energy. MD simulations (200 ns) of the predicted structures were performed using the Groningen Machine for Chemical Simulations (GROMACS) [61]. The final frames of the structures after the 200 ns MD simulations are depicted in Figure 5. All models folded into compact 3D structures were compared with the conformations before MD simulations (Figure 1). The structures modeled using the neural network-based tools exhibited large-scale changes during MD simulations, from relatively extended states to compact folded states. We further determined the root mean square deviation (RMSD) of backbone atoms, radius of gyration (Rg), and root mean square fluctuation (RMSF) of Cα atoms (α-carbons) of the structures over 200 ns to analyze the stability of the predicted models and the compactness of the structures.

**Figure 6:**
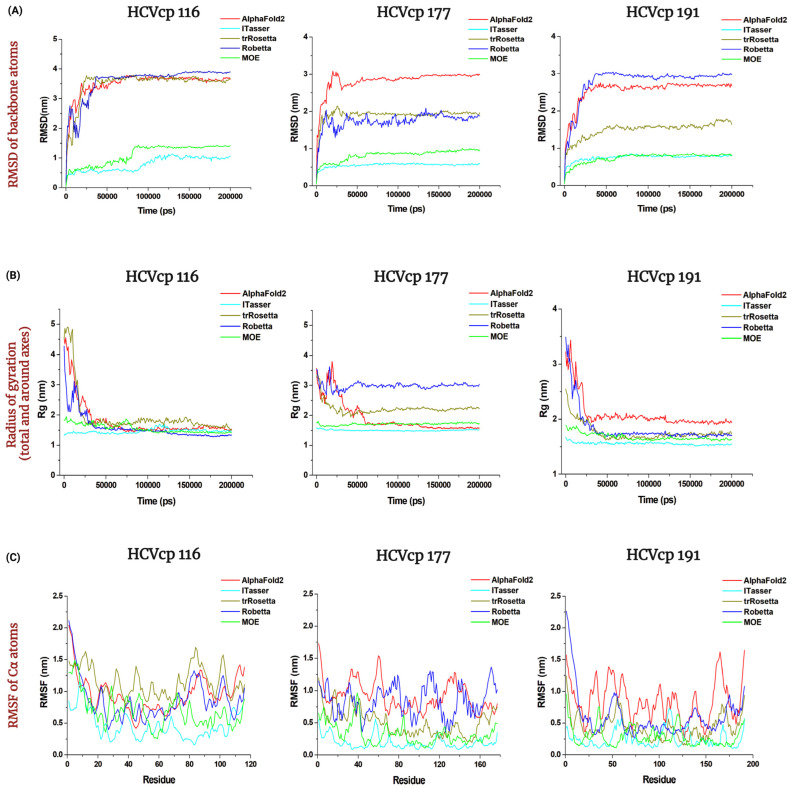
MD simulation trajectories of (**A**) RMSD of backbone atoms, (**B**) radius of gyration, and (**C**) RMSF of Cα atoms for the models of HCVcp 116, HCVcp 177, and HCVcp 191 during the 200 ns MD simulations. The red, blue, dark yellow, green, and cyan lines indicate the values for the models constructed using AF2, Robetta, trRosetta, MOE, and I-TASSER, respectively.

The MD simulation trajectories of the models are presented in Figure 6. For HCVcp 116, the models constructed using the neural network-based tools exhibited large structural changes during MD simulations, as evident from the plots corresponding to the RMSD of backbone atoms. The RMSD values increased by approximately 3.5 nm during the first 50 ns and then plateaued, indicating that the structures had reached a stable state. By contrast, the models constructed using the template-based tools exhibited small-scale structural changes during MD simulations. The RMSD values increased by approximately 1–1.5 nm, and the structures required approximately 100 ns to reach a stable state. For HCVcp 177, the model constructed using AF2 exhibited the largest increase (approximately 3 nm) in the RMSD of backbone atoms during MD simulations. The models constructed using Robetta and trRosetta exhibited an increase of approximately 2 nm in the RMSD of backbone atoms. This value increased by 1 and 0.5 nm during the MD simulations of the models constructed using MOE and I-TASSER, respectively. For HCVcp 191, the increases in the RMSD of backbone atoms during MD simulations were approximately 3, 2.5, and 1.5 nm for the models constructed using Robetta, AF2, and trRosetta, respectively. Notably, the models constructed using MOE and I-TASSER exhibited relatively small changes in the RMSD of backbone atoms (an increase of <1 nm). According to the trajectories, all the HCVcp 191 models reached a stable state within less than 75 ns.

Rg, the root mean square distance of the parts of an object from either its center of mass or a given axis, can be used as an indicator of the compression behavior or compactness of an object. This measure can also be used to determine the compactness of proteins [62]. As shown in Figure 6, the Rg values of the HCVcp 116 models constructed using AF2, Robetta, and trRosetta decreased from approximately 4.5 nm to approximately 1.5 nm during MD simulations, indicating that these models folded from relatively loose structures to compact ones. The Rg values of both of the models constructed using MOE and I-TASSER were approximately 1.5 nm, which remained relatively constant throughout MD simulations. For HCVcp 177, the Rg of the models constructed using AF2 and trRosetta decreased from approximately 3.5 nm to 1.5 and 2 nm during MD simulations, respectively. Only a small reduction from 3.5 nm to 3 nm was observed in the Robetta-predicted model during the simulations. The Rg values of the models constructed using MOE and I-TASSER remained constant at approximately 1.5 nm during the simulations. For HCVcp 191, the Rg values of the models constructed using Robetta, AF2, and trRosetta decreased from 3.5 nm to approximately 1.75 nm, from 3.5 nm to approximately 2 nm, and from 2.5 nm to approximately 1.75 nm, respectively. By contrast, the models constructed using MOE and I-TASSER exhibited small changes in Rg values during the simulations. The Rg values of the models constructed using MOE and I-TASSER remained constant (approximately 1.5–1.75 nm) during the simulations. When the structures of 150, 175, and 200 ns frames in MD simulations for all models are superimposed, it can be seen that the structures have reached their stable state in MD simulations (Supplementary Figure S7).

The RMSF of Cα atoms indicates the fluctuations in each Cα atom of a protein backbone; thus, this value can measure the movement of each Cα atom in simulation studies. The RMSF values of Cα atoms obtained for the models constructed in our study are presented in Figure 6. Among the models constructed for HCVcp 116, the model predicted using trRosetta exhibited the highest level of fluctuations along its primary structure in the simulations, whereas that predicted using I-TASSER exhibited the lowest level of fluctuations. For HCVcp 177, the model predicted using AF2 exhibited the highest level of fluctuations, whereas those predicted using MOE and I-TASSER exhibited considerably lower levels of fluctuations. For HCVcp 191, the model predicted using AF2 exhibited the highest level of fluctuations, whereas those predicted using MOE and I-TASSER exhibited markedly low levels of fluctuations in Cα atoms.

### 3.5. Results of Refined Structure Analysis

**Figure 7:**
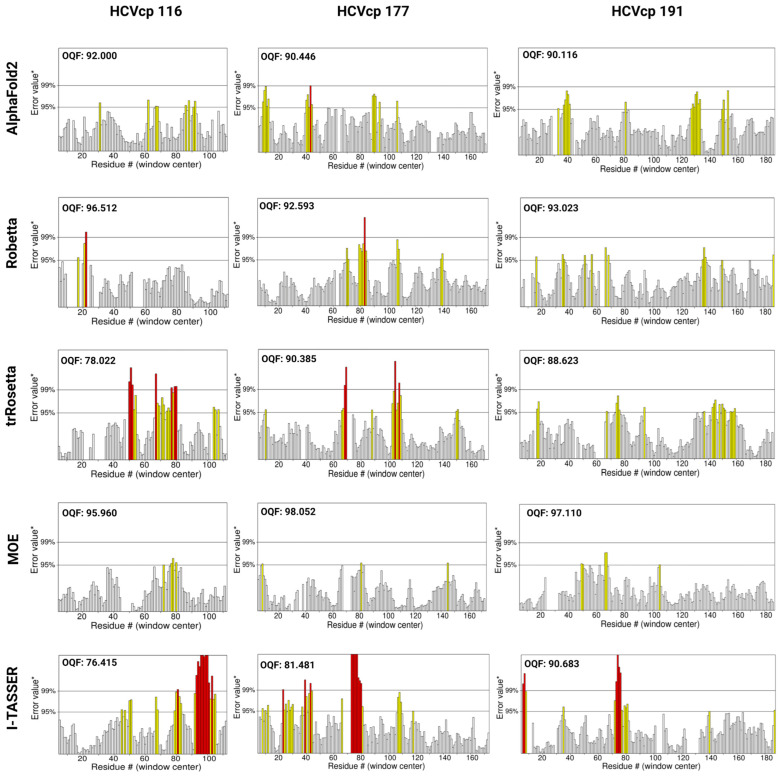
ERRAT plots for the refined HCVcp 116, HCVcp 177, and HCVcp 191 models. The models were predicted using AF2, Robetta, trRosetta, MOE, and I-TASSER and subsequently subjected to 200 ns MD simulations. The plots show two lines (on the error axis), which indicate the confidence level at which regions exceeding the error value can be rejected. The regions that can be rejected at the 95% confidence level are in yellow, whereas those that can be rejected at the 99% confidence level (99% confidence in being erroneous) are in red. OQF, overall quality factor.

The ERRAT plots for the refined structures are depicted in Figure 7. For all the models constructed using AF2, considerable reductions were noted in erroneous regions compared with the results shown in Figure 3; these reductions indicated substantial improvements in the quality and reliability of the refined models. The models constructed using MOE had no region that could be rejected with 99% confidence (no red region), suggesting the good quality of the models. The HCVcp 116 and HCVcp 177 models constructed using Robetta and trRosetta still contained a few erroneous regions. Despite refinement-based improvements, erroneous (red) regions were noted in Domain 1 of the models constructed using I-TASSER. The most problematic region in the HCVcp 116 model constructed using I-TASSER was the region between AA 93 and AA 105. For HCVcp 177 and HCVcp 191, the problematic regions were common and were located between AA 70 and AA 80.

MD simulations also improved the quantitative scores of the modeled structures. The scores are presented in Figure 7. Compared with the corresponding pre-refinement scores, substantial improvements were noted in most OQF scores of the HCVcp 116, HCVcp 177, and HCVcp 191 models constructed using AF2, Robetta, trRosetta, and I-TASSER. The largest increase in ERRAT scores after MD simulations was observed for the models constructed using AF2. All three AF2-constructed refined models had an ERRAT OQF score > 90. However, even after refinement, the HCVcp 116 models constructed using trRosetta and I-TASSER had an OQF score < 80.

**Figure 8:**
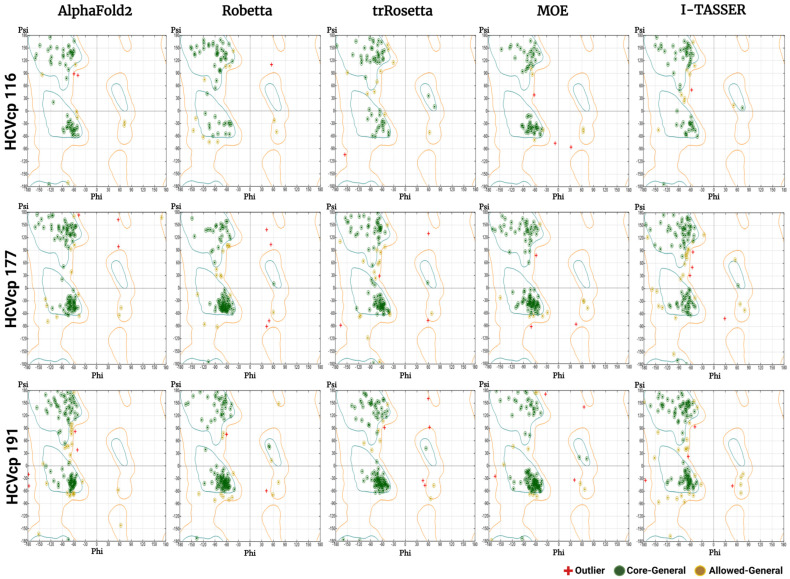
Phi–psi plots of the refined HCVcp 116, HCVcp 177, and HCVcp 191 models. The models were constructed using AF2, Robetta, trRosetta, MOE, and I-TASSER and subsequently subjected to 200 ns MD simulations. The cyan outline corresponds to the most favored regions (conformations with no steric hindrance). The orange outline corresponds to the allowed regions. The regions outside the orange outline are disallowed regions (sterically forbidden regions).

Phi–psi plots were generated for the refined models (Figure 8). The HCVcp models constructed using AF2 and I-TASSER initially had poor quality. However, after MD simulations, the phi–psi plots indicated considerable improvements in the quality of all three models constructed using these two tools. The number of AAs in the disallowed region decreased significantly. Most AAs of the structures were found in the most favored regions of the phi–psi plots.

### 3.6. Results of Structural Validation

**Figure 9:**
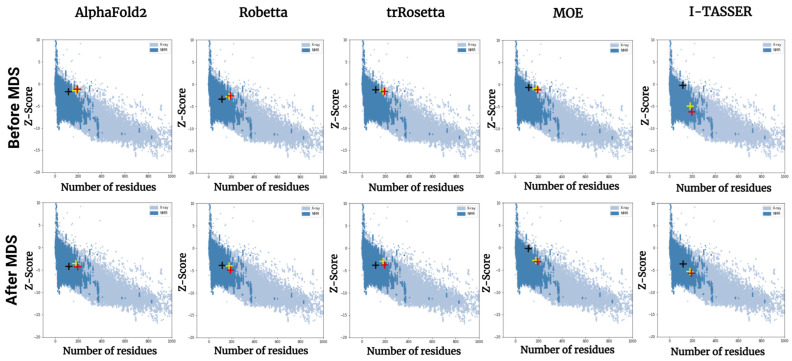
Results of the ProSA-web-based validation of all HCVcp models before (upper panels) and after (lower panels) MD simulations. The ProSA-web z-scores of all available PDB protein structures determined through X-ray crystallography (light blue) or NMR spectroscopy (dark blue) with respect to length. The black, yellow, and red crosses indicate the z-scores of the HCVcp 116, HCVcp 177, and HCVcp 191 models, respectively. ProSA, Protein Structure Analysis.

The Protein Structure Analysis (ProSA)-web [45] is a tool for identifying potential errors in the 3D models of proteins. The ProSA-web z-score of a structure indicates the overall model quality and measures the deviation of the structure’s total energy from the energy distribution derived from random conformations. The overall quality score for a query structure analyzed using ProSA-web is displayed in a plot, which shows the scores of all available PDB protein structures determined experimentally through X-ray crystallography and NMR spectroscopy [45,69]. Protein structures with z-scores outside the range for typical protein characteristics are regarded as erroneous structures. The ProSA-web scores of the initial and refined models are presented in Figure 9. Even before MD simulations, all models had z-scores (Supplementary Table S3) within the range of scores typically observed for native proteins of similar size. After MD simulations, improvements were noted in the scores of the refined models (Supplementary Table S4). The z-scores of the refined models, except the HCVcp 116 model predicted using MOE, moved toward the center of the z-score range of native proteins of similar size.

## 4. Discussion

The 3D structures of biomolecules are essential for the analysis of basic biological functions, structural changes in mutant proteins, interactions between biomolecules, and biological complex assembly. Knowledge obtained through the structural analysis of proteins may be directly applied to the development of effective treatment strategies for various diseases. Traditionally, the 3D structures of proteins have been obtained experimentally using techniques such as X-ray crystallography, NMR spectroscopy, and cryogenic electron microscopy. However, the determination of protein structures using these techniques may not always be successful. X-ray diffraction data can only be obtained from high-quality crystals [70], but not all proteins can be successfully crystallized. NMR spectroscopy has drawbacks such as a sensitivity to experimental conditions, the need for highly soluble samples, and signal overlaps [71,72]. In the absence of suitable samples and conditions, protein structures cannot be obtained. The major limitations of electron microscopy are its dependence on large quantities of purified protein samples, which can be difficult to obtain for some proteins, and the difficulty in interpreting the obtained images [73,74]. With advancements in computer technology, in silico methods have emerged as powerful tools in biomedical research. Computational methods such as molecular docking and MD simulations have enabled studies on protein–protein and ligand–receptor interactions, thus advancing research on drug development. Computational tools have been developed for the efficient modeling of protein structures from their AA sequences without the need for performing wet-laboratory experiments. The traditional method for computing the structures of target proteins is called homology modeling, which requires experimentally resolved template structures of the target protein’s homolog. To generate reliable protein structures, structural templates with >40% sequence identity to the target protein are needed [60]. However, for some proteins, particularly viral proteins, suitable templates for homology modeling are not available. Thus, tools that predict protein structures without the requirement for templates are crucial. According to Anfinsen’s dogma, the native structure of a protein is determined solely on the basis of its AA sequence [75,76]. Therefore, structural models of proteins can be constructed directly from their AA sequences. Tools have been developed for this purpose. These tools often adopt neural network-based deep learning approaches [77], in which the systems are trained with data from the PDB. In the present study, we used both neural network-based tools (AF2, Robetta, and trRosetta) and template-based tools (MOE and I-TASSER) to construct 3D models corresponding to varying lengths of HCVcp. The models constructed using different tools were compared and analyzed. Considering the lack of suitable templates for modeling the entire HCVcp structure, for template-based MOE modeling, we modeled HCVcp domains/segments separately and then assembled them to obtain the complete structure of the protein. According to ERRAT analysis results, the models constructed using Robetta exhibited the highest quality, followed by those constructed using trRosetta (Figure 3). By contrast, the quality of the initial models predicted using AF2 was poor. Regarding template-based modeling tools, MOE outperformed I-TASSER. The satisfactory quality of the models constructed using Robetta, trRosetta, and MOE was also reflected by the corresponding phi–psi plots (Figure 4). However, the phi–psi plot analysis provided poor results for the models predicted using AF2 and I-TASSER. The results of secondary structure prediction revealed that HCVcp Domains 2 and 3 contain several α-helices. These α-helices were observed in all HCVcp 177 and HCVcp 191 models, except for those constructed using I-TASSER. Secondary structures, which are mainly formed through hydrogen bonding, are relatively easy to predict. AAs can be grouped into categories that are likely to form α-helices, β-strands, turns, and coils. Predicted secondary structures often exhibit high levels of consistency with high-resolution experimental structures [78,79,80]. Therefore, the secondary structures of proteins can be used to validate predicted 3D models. Interestingly, although the ERRAT scores of the trRosetta models were lower than those of the Robetta and MOE models, the β-strand structures in HCVcp Domain 1 were only predicted using trRosetta. Some of the predicted structures were not acceptable according to the validation results; thus, further refinement was performed. MD simulation is a feasible strategy for the refinement of protein structures [68,81]. Therefore, all models constructed in our study were subjected to MD simulations. The fundamental theory is that given sufficient time, a protein structure will reach a stable state with the lowest free energy. All models constructed using the neural network-based tools folded into relatively compact structures during the simulations (Figure 5). After refinement, the models predicted using trRosetta exhibited the highest levels of agreement with the results of secondary structure prediction (Figure 2 and Figure 5). In ERRAT analysis, a score of >75 was obtained for all refined models, and a score of >90 was obtained for 11 of 15 models (Figure 7). On the basis of the ERRAT analysis results, the models constructed using AF2, Robetta, and MOE exhibited the highest quality: with a score of >90 being obtained for all predicted structures. After MD simulations, the models constructed using AF2 exhibited considerable improvements, as evident from the results of ERRAT and phi–psi plot analyses. These findings indicate that structural refinement through energy minimization can enhance the reliability of structural models predicted using AF2.

Efficient tools for the construction of protein models directly from their AA sequences are crucial for ensuring advances in biomedical research and for accelerating the development of cost-effective treatment strategies for various diseases. Substantial progress has recently been made in protein structure predictions, particularly de novo template-less predictions. This progress can mostly be attributed to the application of deep learning techniques, which have become indispensable approaches for improving the accuracy of protein structure predictions. In this study, we compared three popular neural network-based techniques, AF2, Robetta, and trRosetta, in terms of their efficiency for predicting the structures of varying lengths of a viral protein. AF2 exhibited an excellent performance in CASP experiments; this tool accurately predicted the structures of most proteins in the assessment [9]. However, in our study, AF2 performed poorly in the initial predictions. Robetta and trRosetta outperformed AF2. Similar differences between these tools have been observed in other studies. Lee et al. [13] compared the protein structures predicted using AF2, RoseTTAFold, and a template-based modeler with the crystal structure of rhodopsin bound to visual arrestin (PDB 5W0P); the structure predicted using AF2 varied considerably from those predicted using the other tools, which exhibited high levels of agreement with the crystal structure. Azzaz et al. [82] predicted the structures of several proteins using computational tools and compared the obtained models with the corresponding crystal structures; they found that Robetta was generally more accurate than AF2. Together with our results, these findings suggest that structural refinement is required to improve the quality of the models predicted using AF2. Regarding template-based tools, we found that MOE, for which templates were selected manually, outperformed I-TASSER, which automatically selected the suitable templates. We performed 200 ns MD simulations to refine the predicted models. The models reached stable states within 100 ns. After refinement, considerable improvements were noted in the scores of the models predicted using AF2 and I-TASSER; the refined structures became theoretically reasonable according to the results of ERRAT and phi–psi plot analyses. Post-refinement improvements in model quality were also indicated by the ProSA-web analysis [45], which calculated the energy values of the models, and the values were compared with those of typical native folded protein structures (Figure 9). Therefore, structural refinement through thermodynamic stabilization may be essential for the construction of reliable protein models. Furthermore, MD simulation is a feasible approach for this purpose.

It is worth noting that for proteins with intrinsically disordered regions, such as HCVcp [83], obtaining theoretically correct structures without a steric clash is just the first step. It is challenging for these in silico predicted structural models with disordered regions to be validated without directly relevant data from experimental techniques, such as X-ray crystallography and NMR. For structure-based investigations, the unvalidated predicted structural models of disordered-region-containing proteins should be used with caution. Further works can be performed by using Markov state modeling [84] and enhanced sampling MD simulation methods [85], such as replica exchange MD [86] and metadynamics [87], to sample different protein conformations for describing the proteins as structural ensembles, in particular for proteins containing disordered regions.

## Data Availability

The data presented in the present study are available from the corresponding author upon reasonable request.

## Supplementary Materials

The following supporting information can be downloaded at: https://www.mdpi.com/article/10.3390/bioengineering10091004/s1, Supplementary Figure S1: Pictorial alignment of NCBI-BLASTp results used for MOE-based structure prediction; Supplementary Table S1: Top ten templates automatically selected by I-TASSER for HCVcp structural modeling; Supplementary Table S2: Summary of the simulation systems in this study; Supplementary Figure S2: Water boxes containing AF2 predicted HCVcp 116, HCVcp 177, or HCVcp 191 structures for MD simulations; Supplementary Figure S3: Water boxes containing Robetta predicted HCVcp 116, HCVcp 177, or HCVcp 191 structures for MD simulations; Supplementary Figure S4: Water boxes containing trRosetta predicted HCVcp 116, HCVcp 177, or HCVcp 191 structures for MD simulations; Supplementary Figure S5: Water boxes containing MOE modeled HCVcp 116, HCVcp 177, or HCVcp 191 structures for MD simulations; Supplementary Figure S6: Water boxes containing I-TASSER modeled HCVcp 116, HCVcp 177, or HCVcp 191 structures for MD simulations; Supplementary Figure S7: Superimposed structures of 150, 175, and 200 ns frames in MD simulations for all the models; Supplementary Table S3: Validation of the predicted HCVcp structures; Supplementary Table S4: Validation of the predicted HCVcp structures subjected to a 200 ns MD simulation.
