# Supplementary material for: Comparison, Analysis, and Molecular Dynamics Simulations of Structures of a Viral Protein Modeled Using Various Computational Tools
Source: Bioengineering (Basel). 2023 Aug 24;10(9):1004. doi: 10.3390/bioengineering10091004 (PMC10525864; doi:10.3390/bioengineering10091004)
Supplement: Supplementary file 1 [file bioengineering-10-01004-s001.zip › bioengineering-2516384-supplementary.pdf]

Supplementary Information

Comparison, Analysis, and Molecular Dynamics Simulations of Structures of a Viral Protein Modeled Using Various Computational Tools

Hemalatha Mani [1], Chun-Chun Chang [2,3], Hao-Jen Hsu [4], Chin-Hao Yang [5], Jui-Hung Yen [6] and Je-Wen Liou [1,3,5,*]

1 Institute of Medical Sciences, Tzu Chi University, Hualien 97004, Taiwan
2 Department of Laboratory Medicine, Hualien Tzu Chi Hospital, Buddhist Tzu Chi Medical Foundation, Hualien 97004, Taiwan
3 Department of Laboratory Medicine and Biotechnology, Tzu Chi University, Hualien 97004, Taiwan
4 Department of Biomedical Sciences and Engineering, Tzu Chi University, Hualien 97004, Taiwan
5 Department of Biochemistry, School of Medicine, Tzu Chi University, Hualien 97004, Taiwan
6 Department of Molecular Biology and Human Genetics, Tzu Chi University, Hualien 97004, Taiwan
* Correspondence: jwliou@mail.tcu.edu.tw

A

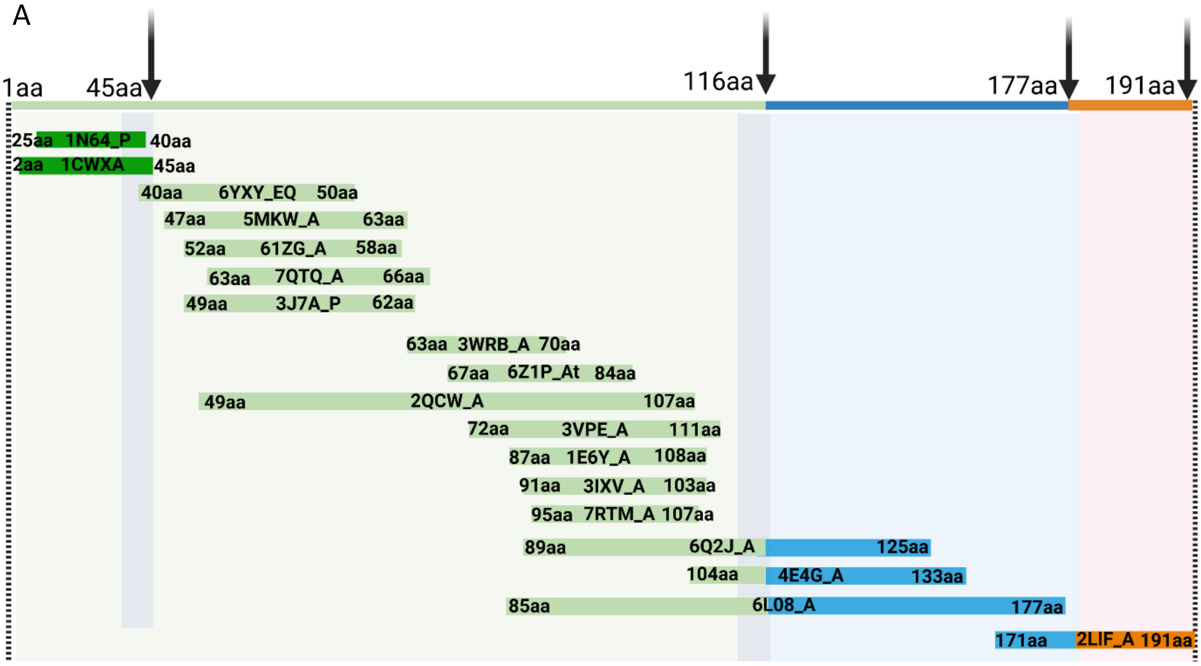

B

PDB Code	UniProt ID	Sequence Alignment
1CWX	P27958-1	>Query 2 STNPKPQRKTKRNTNRRPQDVKFPGGGQIVGGVYLLPRRGPRLG 45<br>STNPKPQRKTKRNTNRRPQDVKFPGGGQIVGGVYLLPRRGPRLG<br>>1CWX_A STNPKPQRKTKRNTNRRPQDVKFPGGGQIVGGVYLLPRRGPRLG 44
1N64	P29846	> Query 25 PGGGQIVGGVYLLPRR 40<br>PGGGQIVGGVYLLPRR<br>>1N64_P 1 PGGGQIVGGVYLLPRR 16
6YXY	Q380Y8	>Query 40 RGPRLG----VR-TTR 50<br>R PRLG VR TTR<br>>6YXY_EQ 98 RNPRLGRAGRVRGTTR 113
5MKW	Q5FWF4	>Query 47 RTTRKTSERSQPRGRRQ 63<br>RT R ERSQ RRQ<br>>5MKW_A 103 RTARQAKERSQ--VRRQ 117
6IZG	Q57UL0	>Query 52 TSERSQP 58<br>TSERSQP<br>>6IZG_A 22 TSERSQP 28
7QTQ	P01267	>Query 63 QPIP 66<br>QPIP<br>>7QTQ_A 710 QPIP 713
3J7A	NA	>Query 49 TRKTSERSQPRGRR 62<br>TRK S R RGRR<br>>3J7A_P 140 TRKKSGR---RGRR 150
3WRB	G2IKE5	>Query 63 QPIPKARR 70<br>+PIPKARR<br>>3WRB_A 154 HPIPKARR 161
6Z1P	I7M2I9	>Query 67 KARRPEGRTWAQPGYPWP 84<br>K+R+P+ W P Y WP<br>>6Z1P_At 54 KSRKPKQVKWDDPNYTWP 71
2QCW	P22004	>Query 49 TRKTSERSQPRGRRQPIPKARRPEGRTWAQPGYPWPLYGNEGCGWAGWLLSPRGSRPSW 107<br>+R S +SQ R E +T + + + + GW W+++P+G ++<br>>2QCW_A 3 SRNRSTQSQDVARVSSASDYNSSELKTACRKHELYVSF--QDLGWQDWIIAPKGYAANY 59
3VPE	G5ELM3	>Query 72 EGRTWAQPGYPWPLYGNE---GCG--WAGWLLSPRGSRPSWGPTD 111<br>+ R W+ P P+ +YGN G G A L SP+G G T+<br>>3VPE_A 1 QDRDWSSPQQPFTIYGNTHYVGTGGISAVLLSSPQGHILVDGTTE 45
1E6Y	P07962	>Query 87 GNEGCGWAGWLLSPRGSRPSWG 108<br>GN G +GW LS + +WG<br>>1E6Y_A 437 GNANAGLSGWYLSMYLHKEAWG 458
3IXV	P80476	>Query 91 CGWAGWLLSPRGS 103<br>CGW +L PRGS<br>>3IXV_A 533 CGWPEHMLIPRGS 545
7RTM	Q6RVG2	>Query 95 GWLLSPRGSRPSW 107<br>GW +SP G P W<br>>7RTM_A 319 GWFISPIGPNPWW 331
6Q2J	Q99988	>Query 89 EGCGWAGWLLSPRGSRPSW--GPTDPRRRSRNLGKVIDT 125<br>E GWA W+LSPR + + G + R+ N+ I T<br>>6Q2J_A 48 EDLGWADWVLSPREVQVTMCIGACPSQFRAANMHAQIKT 86
4E4G	Q92RW4	>Query 104 RPSWGPTDPRRRSRNLGKVIDTLTCGFADL 133<br>+P W T+P+RR+R K + L +L<br>>4E4G_A 77 QPKWAATNPQRRARVFXKFVQLLNDNXNEL 106
6L08	O65896	>Query 85 LYGNEGCGWAGWLLSPRGSRPSWGPTDPRRRSRNLGKVIDTLTCGFADLMGYIPLVGAPL 144<br>L +G + GW + PS GP ++D + G GY +VGA L<br>>6L08_A 218 LVDCDGKVYRGWYMESAAYNPSMGPVQ--------AALVDYVANGGGG--GYERIVGAVL 267<br>>Query 145 GGAARALA---HGVRVLEDGVNYATGNLPGCSFSIF 177<br>A+ H R+L + ++ P C F +F<br>>6L08_A 268 VEKEDAVVRQEHTARLLLETIS------PKCEFKVF 297
2LIF	Q99IB8	>Query 171 GCSFSIFLLALLSCLTVPASA 191<br>G FSIFLLALLSC+TVP SA<br>>2LIF_A 3 GFPFSIFLLALLSCITVPVSA 23

Supplementary Figure S1. Pictorial alignment of NCBI-BLASTp results used for MOE-based structure prediction. (A) Aligned positions and PDB codes of the structural templates. The domain 1 from AA 1 to AA 116, domain 2 from AA 117 to AA 177 and domain 3 from AA 178 to AA 191 are colored in green, blue, and red, respectively. 1N64 and 1CWX at the N-terminal and 2LIF at the C-terminal are PDB available partial structures for HCVcp. (B) PDB codes, UniProt IDs, and sequence alignments of the structural templates.

Supplementary Table S1. Top ten templates automatically selected by I-TASSER for HCVcp structural modeling.

	HCVcp 116		HCVcp 177		HCVcp 191	
	Threading alignments		Threading alignments		Threading alignments	
No.	PDB	Zi-score	PDB	Zi-score	PDB	Zi-score
1	1xcq	6.08	1xcq	4.47	1xcq	4.43
2	7jjvA	1.95	1cwxA	0.45	1xcq	3.54
3	1xcq	4.16	7l6yA	0.79	1xcq	5.05
4	3hd4	1.03	1xcq	3.43	1xcqQ	1.57
5	1cwxA	2.24	1cwxA	1.09	1cwxA	1.72
6	6em3V	1.53	1xcq	5.08	1cwx	4.43
7	1xcq	5.32	1xcqQ	1.54	1cwx	3.54
8	1cwxA	0.94	1cwxA	1.02	1cwx	5.05
9	7nycA	0.80	7wooD	0.84	1xct	2.91
10	1xcqQ	1.57	1qy6A	0.61	1xct	2.33

I-TASSER identifies structural templates from the PDB by a multiple threading approach. Zi-score in this table is the normalized difference between the raw and average scores of standard deviations for the threading alignments, Zi-score >1 is represents a good alignment and vice versa.

Supplementary Table S2. Summary of the simulation systems in this study.

Tools	Simulation system	Simulation time (ns)	Total number of atoms	Number of sodium atoms	Number of chloride atoms	Simulation box size (Å)
AlphaFold2	HCVcp 116	200	45987	131	153	153 x 76 x 126
	HCVcp 177	200	46691	132	154	116 x 115 x 111
	HCVcp 191	200	46637	132	154	106 x 126 x 110
Robetta	HCVcp 116	200	34688	98	120	83 x 108 x 122
	HCVcp 177	200	30433	87	109	107 x 91 x 100
	HCVcp 191	200	37361	106	128	93 x 136 x 94
trRosetta	HCVcp 116	200	44014	123	145	146 x 118 x 80
	HCVcp 177	200	33520	96	118	122 x 74 x 119
	HCVcp 191	200	36709	105	127	90 x 107 x 122
MOE	HCVcp 116	200	8678	26	48	77 x 66 x 57
	HCVcp 177	200	10393	31	53	84 x 67 x 62
	HCVcp 191	200	14852	44	66	96 x 71 x 71
I-TASSER	HCVcp 116	200	7424	22	44	63 x 65 x 61
	HCVcp 177	200	9093	28	50	65 x 70 x 68
	HCVcp 191	200	9781	29	51	67 x 70 x 70

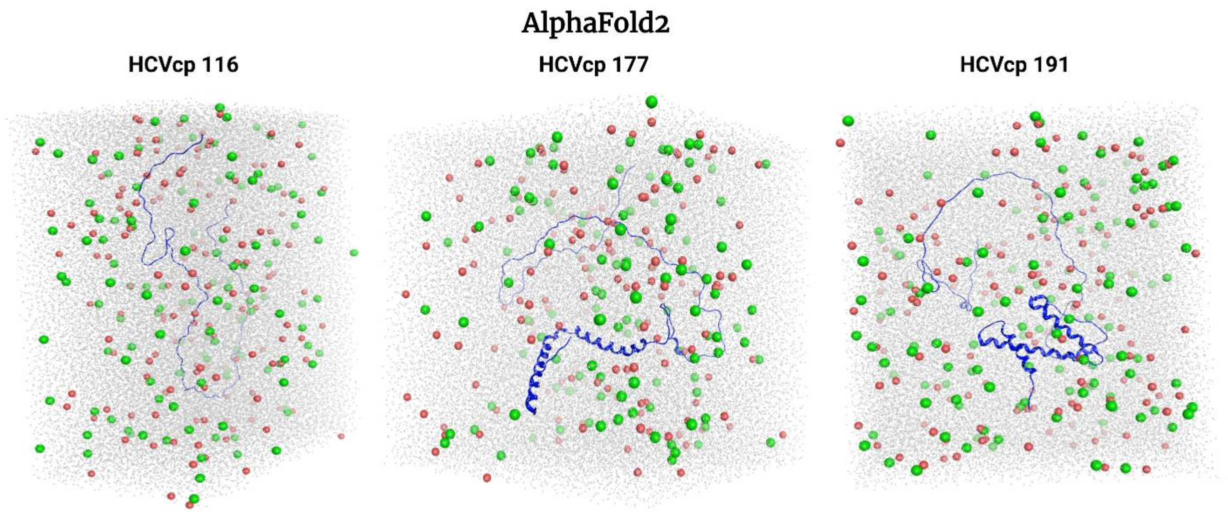

Supplementary Figure S2. Water boxes containing AF2 predicted HCVcp 116, HCVcp 177, or HCVcp 191 structures for MD simulations. In the simulation boxes, the protein structures are colored in blue; water molecules are colored in grey; and sodium and chloride ions are colored in green and red, respectively.

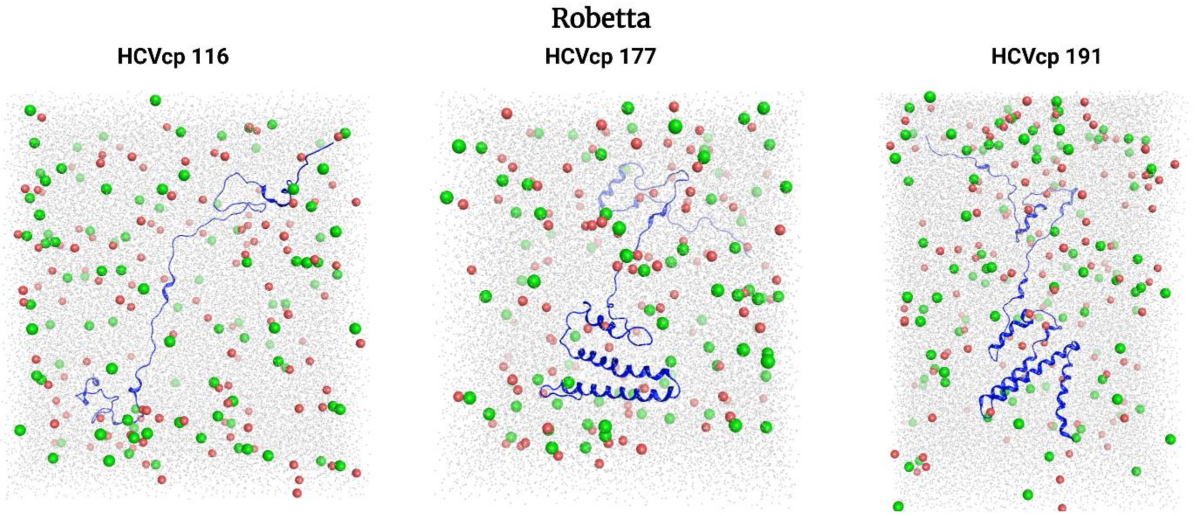

Supplementary Figure S3. Water boxes containing Robetta predicted HCVcp 116, HCVcp 177, or HCVcp 191 structures for MD simulations. In the simulation boxes, the protein structures are colored in blue; water molecules are colored in grey; and sodium and chloride ions are colored in green and red, respectively.

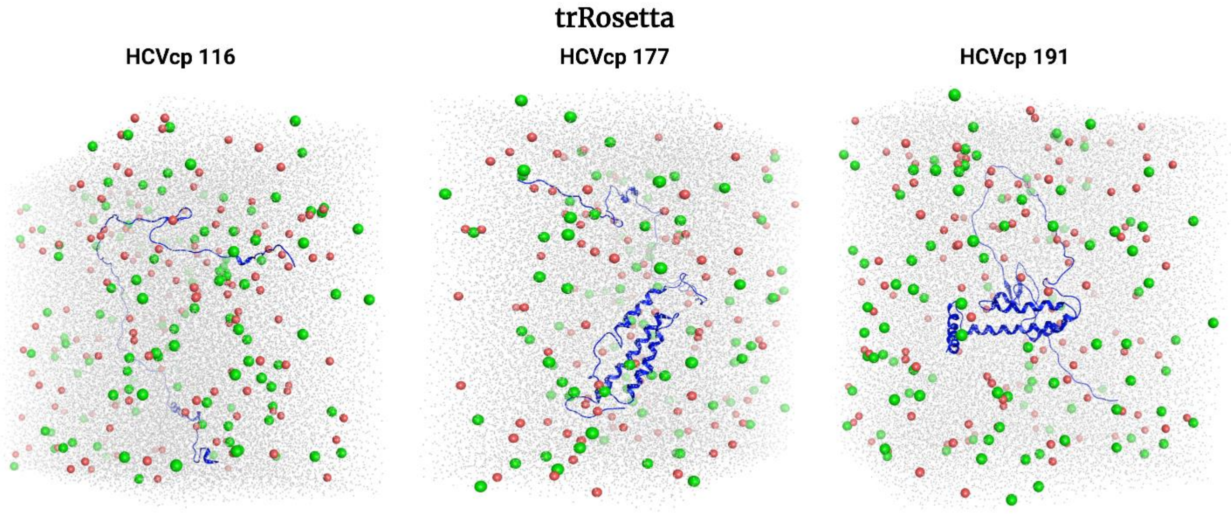

Supplementary Figure S4. Water boxes containing trRosetta predicted HCVcp 116, HCVcp 177, or HCVcp 191 structures for MD simulations. In the simulation boxes, the protein structures are colored in blue; water molecules are colored in grey; and sodium and chloride ions are colored in green and red, respectively.

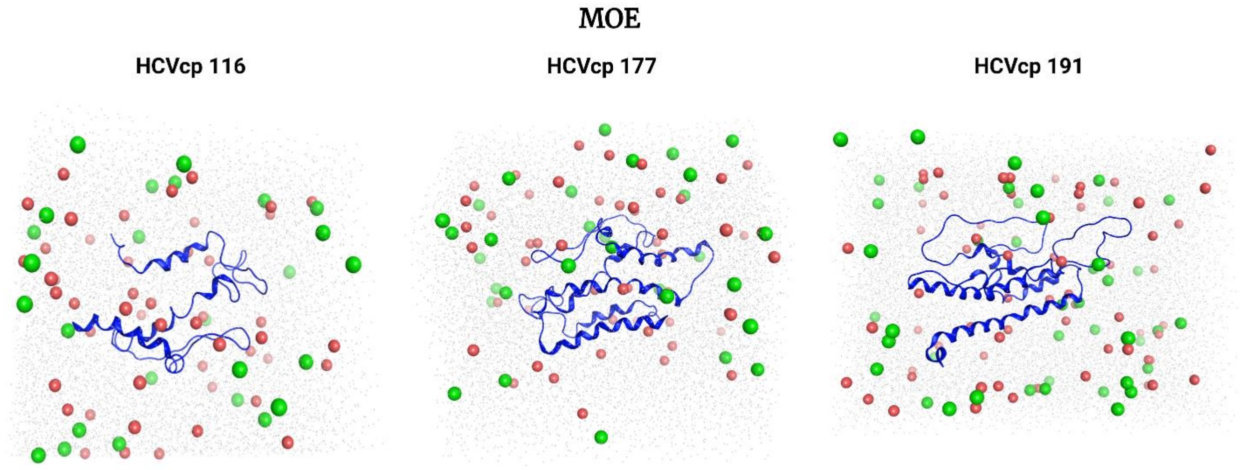

Supplementary Figure S5. Water boxes containing MOE modeled HCVcp 116, HCVcp 177, or HCVcp 191 structures for MD simulations. In the simulation boxes, the protein structures are colored in blue; water molecules are colored in grey; and sodium and chloride ions are colored in green and red, respectively.

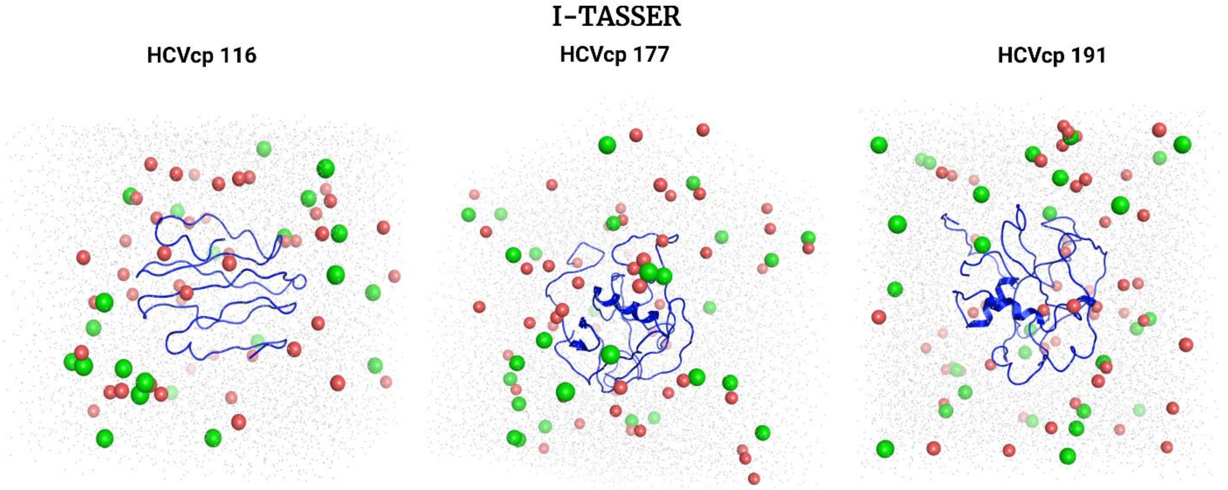

Supplementary Figure S6. Water boxes containing I-TASSER modeled HCVcp 116, HCVcp 177, or HCVcp 191 structures for MD simulations. In the simulation boxes, the protein structures are colored in blue; water molecules are colored in grey; and sodium and chloride ions are colored in green and red, respectively.

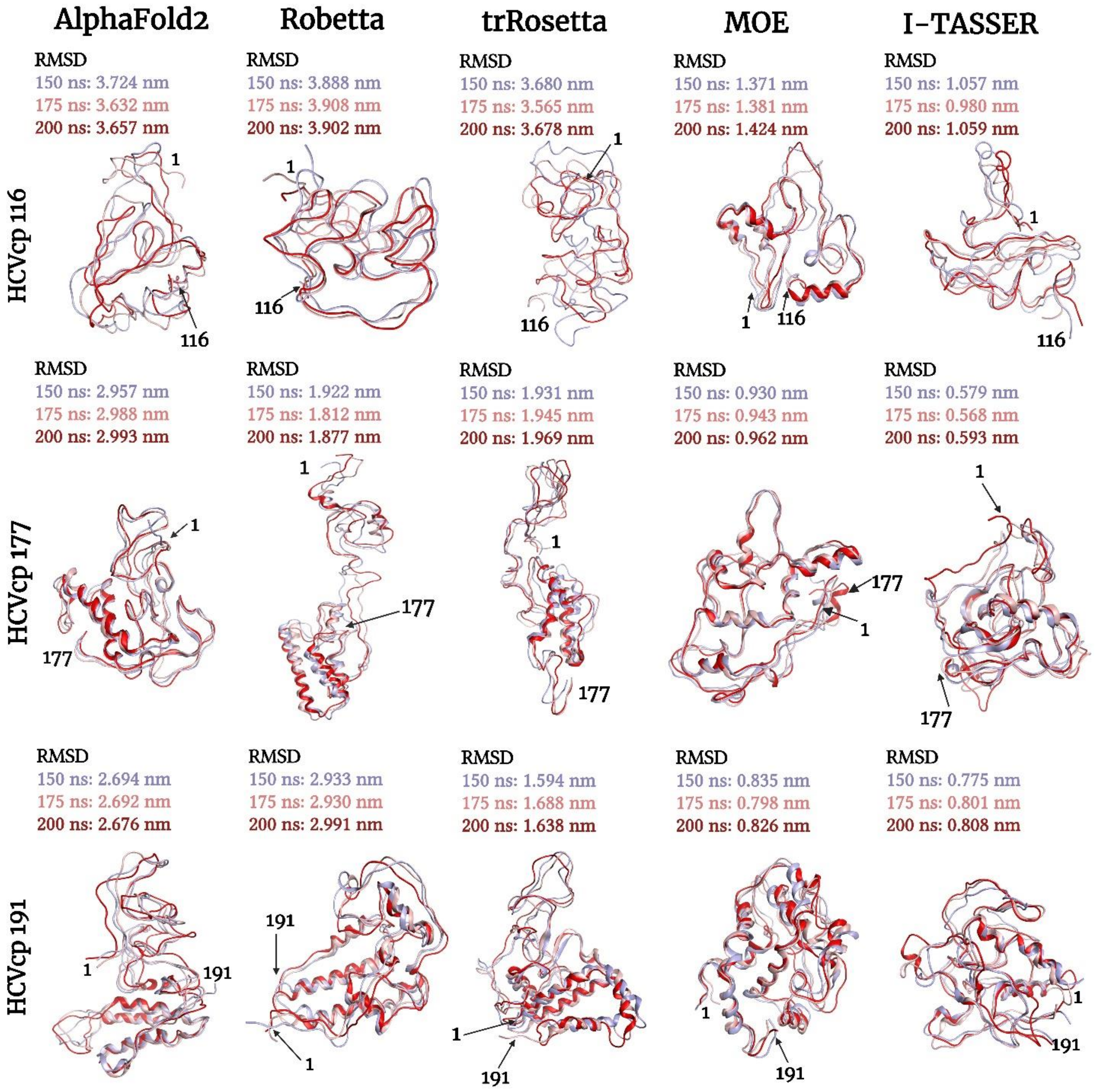

Supplementary Figure S7. Superimposed structures of 150, 175, and 200 ns frames in MD simulations for all the models. As can be seen from the comparison, the structures of 150 (grey), 175 (pink), and 200 ns (red) frames for the models are similar, and the differences (Δ) between RMSD values of 150, 175, and 200 ns structures are small. These results indicate that the structures have reached their stable state.

Supplementary Table S3. Validation of the predicted HCVcp structures.

Modeling tools	HCVcp lengths	ProSA Z-Score
	116	-1.65
AF2	177	-1.50
	191	-1.09
	116	-3.29
Robetta	177	-2.66
	191	-2.56
	116	-1.27
trRosetta	177	-1.60
	191	-1.65
	116	-0.66
MOE	177	-0.82
	191	-1.16
	116	-0.27
I-TASSER	177	-5.03
	191	-6.32

Supplementary Table S4. Validation of the predicted HCVcp structures subjected to 200-ns MD simulation

Modeling tools	HCVcp lengths	ProSA Z-Score
	116	**-4.20**
AF2	177	**-3.57**
	191	**-4.21**
	116	**-3.82**
Robetta	177	**-3.95**
	191	**-4.86**
	116	**-3.76**
trRosetta	177	**-2.97**
	191	**-3.89**
	116	-0.20
MOE	177	**-2.74**
	191	**-2.92**
	116	**-3.58**
I-TASSER	177	**-5.37**
	191	-5.61
